# Efficacy of evidence-based medicine training for primary healthcare professionals: a non-randomized controlled trial

**DOI:** 10.1186/s12909-018-1404-y

**Published:** 2018-12-07

**Authors:** Jiaojiao Fei, Yanhua Li, Weifei Gao, Junwei Li

**Affiliations:** 1 General Practice Department at the Second Hospital affiliated to Zhejiang Chinese Medical University, No. 318 Chaowang Road, Hangzhou, 310000 Zhejiang China; 2 Zhejiang Chinese Medical University, No. 548 Binwen Road, Hangzhou, Zhejiang, Hangzhou, 310000 Zhejiang China

**Keywords:** Evidence-based medicine, Medical education, Primary healthcare

## Abstract

**Background:**

The impact of evidence-based medicine (EBM) training techniques in primary healthcare professionals remains to be determined.

**Methods:**

A non-randomized controlled trial (NRCT) was performed aiming to assess the two methods of evidence-based medicine training for primary healthcare professionals by assessing evidence based practice (EBP) related knowledge (EBP-K), attitude (EBP-A), personal application (EBP-P), anticipated future use (EBP-F), and community management of hypertension. Participants were recruited and assigned to either an EBM training group that receiving a weekly face-to-face EBM training course, or an EBM self-instruction course for eight weeks. A validated instrument was applied to evaluate the four aspects of EBP. Additionally, community management of hypertension was assessed by comparing the the rate of detection, blood pressure control, standard management, grading management and patient satisfaction between 2015 and 2016 to measure training efficacy. The difference between the impact of these two interventions was assessed statistically.

**Results:**

One hundred fifty-one participants (69 in the face-to-face EBM training group and 82 in the self-instruction group) were included. Compared to self-instruction, the face-to-face EBM training was associated with significantly improved EBP-Knowledge (26.14 ± 4.22 vs. 22.44 ± 4.47, *P* < 0.05), EBP-Personal application (22.52 ± 6.18 vs. 16.89 ± 5.99, *P* < 0.05), and EBP-Future use (44.04 ± 8.97 vs. 37.71 ± 8.39, *P* < 0.05). EBP-Attitude scores (10.89 ± 4.52 vs.14.93 ± 5.92, *P* < 0.000) were lower in the EBM training group. Stratified analyses showed that the results were consistent regardless of the participants’ gender, professional role (doctors & apothecaries or nurses), rank (junior or senior doctors & apothecaries), or specialty (Traditional Chinese or Western Medicine). Assessment of community hypertension management revealed that the rate of blood pressure control, standardized hypertension management and patient satisfaction was significantly better in group A than group B (1.14% vs.0.69, 2.85% vs.1.68 and 2.41% vs.0.84%).

**Conclusions:**

A face-to-face EBM training course improved primary healthcare professionals’ EBP knowledge, attitudes, personal application, and anticipated future use. Effective EBM training may improve the efficacy of primary health care services.

**Trial registration:**

Non-Randomized Controlled Trial ChiCTR1800017498, August 1, 2018.

**Electronic supplementary material:**

The online version of this article (10.1186/s12909-018-1404-y) contains supplementary material, which is available to authorized users.

## Background

Evidence based medicine (EBM) is defined as a conscientious, explicit, and judicious use of the current best evidence to inform decisions about the care of individual patients [1]. The positive impact of EBM on clinical practice has been established in healthcare practice all over the world, including in low- and middle-income countries [2–4]. In developed countries, with improved medical education and advanced teaching techniques, great efforts have been made to improve EBM education of medical students and other health care professionals [5–7]. The importance in EBM education is increasingly recognized and efforts have been made to improve the quality of education through the introduction of innovative teaching and training models [4, 8, 9]. It is well accepted that EBM education should be integrated with clinical practice [10], and should be evaluated and guided by evidence of its own effectiveness [11]. Previous research into EBM education and training have demonstrated that EBM knowledge and skills can be improved through various medical school-based education models [12], resident training programs [13] and continuing medical education programs [14]. The training methods employed by these studies typically include extended, self-learning, faculty mentored instruction [12] and a mixture of interactive lectures, workshops and case-based studies [13]. For example, a methodologically sound research conducted in a developing country shows that clinically integrated e-learning EBM curriculum compared with a self-directed EBM course resulted in higher knowledge and skill scores and improved educational environment [3]. Although various strategies, including literature searching education and a blended learning education, have been applied in the teaching of EBM [15–19], the precise impact of these strategies on quality of evidence-based practice (EBP) remain to be determined [20–22].

It has been suggested that EBM curricula should be developed into a 5-step model including translation of uncertainty to an answerable question, systematic retrieval of the best evidence available, critical appraisal of the clinical relevance and applicability of evidence, application of results in practice, and evaluation of performance [2]. Also, it has been noted that EBM educational programs should be designed at an appropriate level for the trainees to optimize learning efficacy [22]. The efficacy of EBM training in undergraduates and residents is established, however EBM training in physicians is less well studied [23]. In mainland China, EBP skills have not traditionally been covered in continuing medical education for primary healthcare practitioners. The optimal strategy for teaching EBM to primary healthcare professionals such as general practitioners (GPs) and nurses, remains to be determined.

Thus, in this study, we carried out an entry-level EBM training program based on the 5-step model for primary health care professionals. The clinical context and specific objectives of the study were:

1. To compare the impact of a face-to-face EBM training strategy and EBM self-instruction on a validated instrument definition of EBP knowledge (EBP-K), attitude (EBP-A), personal application (EBP-P), and future anticipated use (EBP-F), and to develop optimal EBM learning strategies in primary healthcare professionals; and
2. To compare the rate of hypertension management (detection, blood pressure control, hypertension grading, standard management and patient satisfaction) achieved by these two community health service centers.

## Methods

### Trial design

This non-randomized controlled trial was performed with primary healthcare professionals, including doctors, apothecaries and nurses, from the Mi-shi Lane Community Health Service Center and Xiao-he hu-shu Lane Community Health Service Center of Gongshu district in Hangzhou city, and each center has 7 service stations, 106 and 112 medical staffs respectively. Primary healthcare professionals provided basic medical services, prophylactic immunization, women healthcare, and rehabilitation therapy for the community in China. To cater to the professionals working schedule, those who from the same center were allocated to the same group to participate into either a face-to-face EBM training group (group A), or a self-instruction group (group B) by tossing a two-sided digital coin. The participants allocated to group A received a weekly 2-h EBP-structured presentation covering EBP approaches to patient care experiences offered by the EBM faculty team of the second hospital affiliates to Zhejiang Chinese Medical University. The general practice department of the hospital was founded in 2011, and responsible for the tutorship of medical students in the 4th to 5th years of medical school, residents in post-graduate training and continuing medical education for general practice. Participants of group B were assigned to receive a weekly self-instruction course covering the essentials of EBM, which was uploaded by a research assistant through the center’s own network. Blinding and allocation concealment were not possible in the present study because teachers and participants were all aware of the courses they were going to attend. However, study hypothesis had not been disclosed to all participants. This study was based on the provincial fund project, and written informed consent was obtained from each participant. (Additional file 1).

### Participants

Primary healthcare professionals were recruited from two Community Health Service Centers, which have intimate association with the second hospital affiliates to Zhejiang Chinese Medical University. To meet the eligibility, participants were required to be:

1. doctors and apothecaries (including GPs, therapists, apothecaries, public health professionals);
2. nurses;

Participants who were unwilling to participate in the study or did not wish to provide consent were excluded from the study. The following variables were recorded for each participant: age, gender, professional role (doctors & apothecaries or nurses), and rank (junior or senior doctors & apothecaries), or specialty (Traditional Chinese or Western Medicine).

### EBM educational interventions and outcomes

The face-to-face EBM course for group A was developed by a physician and an EBM professor at the second hospital affiliated to Zhejiang Chinese Medical University (Yanhua Li and Junwei Li). This course was intended to provide an interactive forum for participants to improve the clinical implementation of EBM. The primary outcome of this study was knowledge, attitudes, personal application, and anticipated future use, which was measured by using the previously validated EBP-KAB tool [24]. Unlike the medical students, the included primary healthcare physicians had excellent clinical skills, but no formal training in EBM. Thus, the educational intervention was designed as a practical and targeted entry-level EBM training course. The physicians were also extremely busy, so the training schedule was adapted, following feedback, to cater to their work, taking only two hours per week. In total, participants in group A received a 16-h EBM course, including 2 lectures, 3 conferences and 3 small group discussions. The training course is outlined in Table 1. Briefly, the introduction session provided an overview of EBM, including the grading and recommendation of evidence. The subsequent four sessions were developed according to the 5-step model of EBM teaching, covering i) construction of a relevant clinical problem, ii) comprehensive literature search of medical databases, iii) critical appraisal and synthesis of evidence, iv) apply the evidence to the practice. The final session described three actual clinical examples of primary care to demonstrate the real-time application of EBM skills. The EBM faculty team included two professors and three novice teachers. All faculty members participated in a team-based teaching model to develop facilitation and interactive teaching skills to promote the practice of EBM. We also paired experienced teachers with three novice teachers. In these sessions, the latter are encouraged to record trainees’ feedback to the leading teachers. Participant feedback was used to adapt teaching in future sessions. EBM faculty members answered participants’ questions during the training period. For participants in group B, a study assistant would help to upload the same curriculum once a week with documents including texts and pictures via the center’s Office Automation platform, which allows users to transfer data, mail and even voice across the network. Then the participants would download the curriculum file and learn by themselves in their spare time every week, in which circumstances participants finished the mission all by self-discipline or their attitude towards to EBM. Meanwhile, the study assistant was able to see the number of downloads and alert the participants who had not downloaded the files to learn in time. Once the participants had any question, they could communicate with each other or send e-mails to the teachers through the platform, and teachers would make a question list and answer it every week through the platform.

**Table 1:** Evidence-based medicine course curriculum

Topic and learning objectives	Faculty/teaching method/period
Introduction	
1. To describe the definition of EBM	EBM professor (LJW)/lecture/2 h/week
2. To describe the objectives of learning EBM	
3. To describe the principles of practicing EBM	
4. To explain the 5-step model of practicing EBM	
5.To outline the grading and recommendation of evidence.	
Construct a relevant clinical problem	
1. To become familiar with the background problems and the foreground problems	EBM professor (LJW)/conference /2 h/week
2. To construct a foreground problem using PICO method regarding a specific therapy problem	
3. To take excises of constructing relevant clinical problems	
Search literatures	EBM professor (LJW)/Demonstration/ 2 h/week
1. To become familiar with different study types, the best design of studies for answering clinical problems	
2. To become familiar with the category of EBM resources and their strengths and weakness	
3. To improve searching strategies for finding answers to clinical questions	
Statistics terms of EBM	EBM professor (LJW)/Lecture/2 h/week
1.To explain the meaning of relative risk reduction, absolute risk reduction, and number needed to treat	
2. To explain the meaning of sensitivity, specificity, and likelihood ratios and describe how to apply these concepts in clinical decision making	
Assess evidence	EBM professor (LJW)/Conference/2 h/week
1. To determine the relevance between the evidence and the clinical problem	
2. To determine the validity of the evidence	
3. To determine the magnitude and significance of the evidence	
4. To consider patients’ values and perspectives when apply the evidence	
Cases of practicing EBM	EBM faculty (LYH)/ small group discussion/2 h/week
1. a case of hypertension	
2. a case of neck pain	
3. a case of breastfeeding	

In China, the government attaches great importance to the management of hypertension, because, if not treated early, hypertension can become a devastating disease with poor prognosis. Hypertension has been highlighted by the Ministry of health, and in order to improve the management of hypertension, patients’ health records must be available to primary health service professionals. As the management of hypertension is dependent largely on early diagnosis and risk stratification, the case of hypertension in the final session was designed to focus on the application of updated evidence-based hypertension screening, diagnosis and control guidelines, (Chinese Guidelines for the Prevention and Control of Hypertension, 2010 edition). Management of hypertension was assessed by comparing the rate of detection, blood pressure control, standard management, grading management and patient satisfaction between 2015 and 2016. All these five items are included in the patients’ health record, and each item was recorded by medical teams at the service stations at each follow-up.

### Evaluation instrument

Four principle components of EBM, including EBP-K, EBP-A, EBP-P, and EBP-F [24], were measured before and after EBM training using an instrument previously validated for the assessment of EBM education in the undergraduate learning environment [24]. Assessment questionnaires consisted of 26 questions answered using a six point Likert scale. The EBP-K section included five items (scored from 5 to 30), the EBP-A and EBP-P sections six items each (scored from 6 to 36), and the EBP-F section included nine items (scored from 9 to 54) [24] (Additional file 2). These instruments were applied by assessors blinded to participant group to eliminate performance bias.

### Sample size estimation

To estimate the sample size, we assumed that the standard deviation of the two types of intervention was the same when within the two groups, and the probability of alpha = 0.05, beta = 0.10, and the difference between the two increases is 60% of the standard deviation. Known to the delta/sigma = 0.6, the two sides of alpha = 0.05, mu 0.05/2 = 1.282. The input formula is N1 = N2 = 60, so the two groups need 120 people to detect a statistically significant difference. A 10% drop out rate was estimated, so at least 66 participants were required for each group.

### Statistical analyses

All statistical analyses were conducted using SPSS version 17.0. Continuous variables were presented as means and standardized deviations (SDs) if normally distributed; otherwise, median and interquartile range (IQRs) was presented. The categorized variables were presented as numbers and proportions. Differences in continuous variables and categorized variables were analyzed by independent t-test and chi-square test, respectively. Percentage change in scores was compared using the Mann-Whitney test due to their non-normal distribution. Percentage change = (post-course score – pre-course score)/pre-course score *100%. Stratified analyses were preformed according to participants’ gender, professional role (doctors & apothecaries or nurses), and rank (junior or senior doctors & apothecaries), or specialty (Traditional Chinese or Western Medicine). All statistical tests were 2-tailed, and *P* < 0.05 was considered to indicate statistical significance.

## Results

**Fig. 1:**
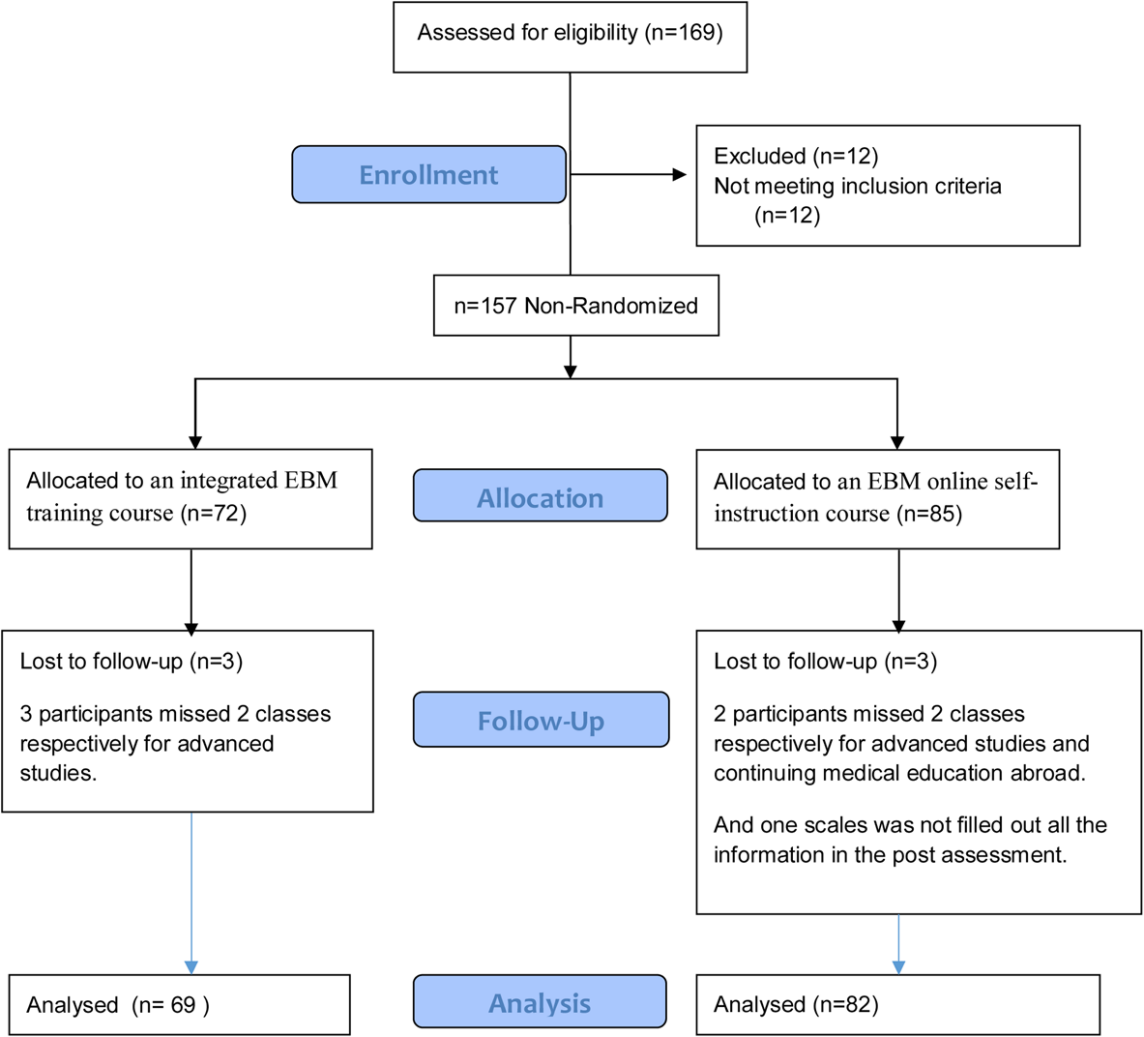
Participant inclusion flow chart

The flow diagram of the study participants throughout the trial is outlined in Fig. 1. Finally, excluding the 12 participants who were not meet the inclusive criteria, we recruited 157 physicians and nurses from these two centers and defined participants from the same center as a “group” for feasibility, and 72 physicians and nurses from Mi-shi Lane center allocated to the trial group (group A), Of those, 69 (95.83%) completed the EBM assessment. Reasons for exclusion included personal attributes leading to lack of timeliness for advanced studies. 85 from Xiao-he Hu-shu Lane center attended to the control group (group B), 82(96.47%) completed the EBM self-learning. Reasons for not completing the exercise included technical difficulties with the online program and not filled out all the information in the post assessment.

Participants’ characteristics such as age, gender, professional role (doctors & apothecaries or nurses), and rank (junior or senior doctors & apothecaries), or specialty (Traditional Chinese or Western Medicine) did not differ significantly between the groups (Table 2).

**Table 2:** Participant Characteristics (chi-square test)

	Structured conference (Group A)	Self-instruction (Group B)	
Characteristics	Group A (*N* = 69)	Group B (*N* = 82)	*P* value
	D&A = 51, Nurse = 18	D&A = 57, Nurse = 25	
Age, years	34.33 ± 7.44	35.45 ± 7.26	0.353
Men (%)	23.19(16)	18.29(15)	0.458
D&A (%)	73.91(51)	69.51(57)	0.551
TCM (%)	37.25(19)	24.56(14)	0.153
Junior D&A (%)	45.10(23)	35.09(20)	0.289

Ps: D&A: Doctors and Apothecaries

EBP was assessed before and after the eight-week course, and changes in the scores for each domain are shown in Table 3, and percentage change in four EBP domains scores in Table 4. For group A, significant changes were observed in the before and after comparisons for all four domains of assessment (all *P* < 0.01) (Table 3). The increase was most pronounced for EBP-P and EBP-K, followed by EBP-A and EBP-F. For group B, consistent results across all four domains of assessment indicated that self-instruction method rarely changed EBP outcomes.

**Table 3:** Percentage change in four EBP domains scores

	Structured Conference, Group A [median (IQR)] (%)	Self-instruction, Group B [median (IQR)] (%)	*P*-value
EBP-K	30.0 (4.5–47.1)	4.2 (−10.0–17.4)	< 0.0001
EBP-A	−25.0 (−58.0–8.0)	3.8 (− 31.6–53.8)	0.0001
EBP-P	60.0 (5.3–141.7)	6.3 (− 14.3–55.6)	0.0002
EBP-F	17.1 (0–35.0)	2.5 (−18.2–19.4)	0.0005

**Table 4:** Assessment of EBM pre- and post- training

	Pre-intervention			Post-intervention		
	Group A	Group B	*P* value	Group A	Group B	*P* value
	*N* = 69	*N* = 82		*N* = 71	*N* = 82	
EBM-K	20.33 ± 4.07	21.56 ± 4.08	0.248	26.14 ± 4.22	22.44 ± 4.47	0.017
EBM-A	16.70 ± 8.76	14.62 ± 5.90	0.086	10.89 ± 4.52	14.93 ± 5.92	0.000
EBM-P	15.36 ± 7.80	15.11 ± 6.65	0.569	22.52 ± 6.18	16.89 ± 5.99	0.000
EBM-F	37.38 ± 7.09	38.09 ± 8.43	0.576	44.04 ± 8.97	37.71 ± 8.39	0.000

Both groups had similar EBP-K, EBP-A, EBP-P and EBP-F scores before the intervention. After the intervention, participants in group A had significantly higher EBP-P (19.52 ± 6.18 vs. 16.89 ± 5.99, *P* < 0.000), EBP-F (44.04 ± 8.97 vs. 37.71 ± 8.39, *P* < 0.000), and EBP-K scores (26.14 ± 4.22 vs. 24.44 ± 4.47, *P* < 0.017) than in with group B. On the other hand, EBP-A scores were significantly lower in group A than B, (10.89 ± 4.52 vs. 14.93 ± 5.92, *P* < 0.000), indicating an improvement in the attitude to EBM.

Stratified analyses showed consistent results across participant characteristics such as gender, professional role, rank, or specialty (Table 5).

**Table 5:** Stratified comparisons of EBM scores after intervention

	Group A				Group B			
	EBM-K	EBM-A	EBM-P	EBM-F	EBM-K	EBM-A	EBM-P	EBM-F
Male	3.12 ± 19.67	16.36 ± 68.09	76.09 ± 100.06	20.56 ± 22.11	2.22 ± 8.61	6.06 ± 23.47	1.72 ± 40.73	2.41 ± 9.34
Female	8.09 ± 24.50	24.37 ± 41.54	53.44 ± 92.25	24.85 ± 36.73	0.52 ± 14.29	2.06 ± 15.92	4.32 ± 29.54	0.19 ± 7.43
*P* value	0.461	0.051	0.447	0.570	0.338	0.594	0.818	0.401
D&A	5.44 ± 24.05	10.83 ± 57.68	60.80 ± 101.32	20.56 ± 34.38	0.82 ± 13.68	3.10 ± 17.89	0.51 ± 24.53	0.40 ± 2.00
Nurse	11.90 ± 25.21	30.01 ± 39.22	61.92 ± 86.12	32.37 ± 31.85	1.94 ± 12.92	3.54 ± 16.66	11.43 ± 43.37	1.04 ± 9.25
*P* value	0.352	0.125	0.964	0.195	0.386	0.914	0.248	0.269
Junior D&A	6.18 ± 19.31	21.73 ± 55.25	61.52 ± 100.55	18.43 ± 32.48	4.11 ± 13.50	2.53 ± 24.64	6.33 ± 31.72	0.36 ± 10.63
Senior D&A	4.83 ± 27.68	1.88 ± 59.07	60.21 ± 103.79	22.30 ± 36.36	0.95 ± 13.62	4.77 ± 22.13	2.63 ± 19.40	1.41 ± 8.55
*P* value	0.839	0.222	0.964	0.690	0.186	0.841	0.259	0.707
TCM D&A	6.67 ± 22.47	9.15 ± 58.36	70.09 ± 68.93	8.18 ± 24.10	6.55 ± 18.54	2.86 ± 7.06	1.53 ± 31.43	2.58 ± 9.67
WM D&A	4.13 ± 20.60	15.79 ± 49.85	59.97 ± 91.39	28.42 ± 38.96	3.22 ± 10.91	6.64 ± 20.55	0.19 ± 22.27	0.53 ± 9.17
*P* value	0.690	0.681	0.880	0.026	0.080	0.197	0.884	0.493

Ps: *TCM* traditional Chinese medicine, *WM* Western medicine

We investigated the management of hypertension as an indirect measure of the clinical effect of EBM training. Our results indicate that the blood pressure control rate, standardized management rate and patient satisfaction was significantly better in group A than group B (1.14% vs.0.69, 2.85% vs.1.68 and 2.41% vs.0.84%) in Table 6. There were no harms or unintended effects in each group.

**Table 6:** Comparison of standardized management and prevention of community hypertension in each group

	Detection rate (%)		Blood pressure control rate (%)		Standard management rate (%)		Grading management rate (%)		Patient satisfaction rate (%)	
	Group A	Group B	Group A	Group B	Group A	Group B	Group A	Group B	Group A	Group B
2015	12.45	14.72	66.01	67.82	68.28	69.33	100	100	96.82	97.13
2016	11.70	13.33	67.15	68.51	71.13	71.01	100	100	99.23	97.97
Percentage change	−0.75	−1.39	1.14	0.69	2.85	1.68	0	0	2.41	0.84

## Discussion

To our knowledge, this is the first study in mainland China to assess the impact of an EBM training program in primary health service professionals using a validated and reliable tool. Our study results indicate that practice based face-to-face training may be an optimal strategy to educate primary healthcare professionals with the application of EBM.

Currently, EBP is recognized as a core competency that must be acquired by all medical professionals, because it can improve the quality of health care by supporting clinical decision-making [25]. Although recent studies have shown that various initiatives may be effective in improving EBM knowledge, no convincing evidence indicates that teaching EBM also changes professional behavior in practice [26]. This study underlines the need not only to enhance EBM skills, but also to improve the ease of use of EBM resources at the point of care. Thus, an entry-level EBM training program was designed by tailoring evidence-based information retrieval systems to busy clinical schedules.

EBM education should be evaluated and guided by evidence of its own effectiveness [27]. Many tools are available to clinicians. And the Fresno test seems to be a key candidate for assessing the efficacy of EBM training [28]. However, it was inappropriate for our participants due to the high item difficulty in the pilot trial. We finally chose a validated instrument for assessment of EBP-K, EBP-A, EBP-P, and EBP-F. Furthermore, application of EBM in clinical practice must be assessed by measuring participants’ care in acute and chronic clinical situations for which there are clear EBM standards [28]. For this reason, we evaluated the management of hypertension by comparing the rate of detection, blood pressure control, standard management, grading management and patient satisfaction between 2015 and 2016. Moreover, this data was available in patients’ health records.

In this study, we found that an eight-week face-to-face EBM training program improved nurse and physician EBP knowledge, attitudes, personal application and anticipated future, and these measures improved more significantly than a self-instruction training program. Subsequent stratified analyses showed that results were consistent regardless of participants’ gender, professional role, rank, or specialty, which indicates that these factors did not affect the efficacy of EBM training. In addition, as assessed the impact of this training on management of community hypertension and found that measures of community hypertension were improved more significantly following face-to-face EBM training than self-instruction training, which indicate that EBM training can significantly improve primary health service staff EBP competency, and contribute to EBP behavior. Lectures, conferences and small group discussions facilitate more interaction between educators and their audience than self-instruction models, suggesting that these interactions may play an important role in EBM training. It might be difficult to gain sufficient EBM knowledge and skills without appropriate guidance by trainers. Face-to-face interaction can provide a superb opportunity for trainees to discuss the problems with professional educators who may provide solutions. The advantage of these methods was observed in all analyzed groups regardless of participants’ gender, professional role, rank, or specialty.

It must be noted that the results of this study may reflect characteristics of particular participants, or the precise training programs applied, and may be limited to interventions of the precise type and duration applied in the current study. In this study, participants were recruited from two community health service centers in Hangzhou city in China. Both sets of participants had the similar EBM background: they had never received formal EBM education but were exposed to EBM through continuing medical education. Second, the EBM training course was designed by EBM professors at the university and teaching hospital, and focused on practical aspects including constructing a clinically relevant question, developing search strategy, assessing the evidence. Third, our training program was implemented for eight weeks. Lengthening or shortening the training duration may change the effects.

### Strengths and limitations

This study has some strength. First, results of our study can minimize the probability of confounding and selection bias to some content, which, nevertheless, were quite common in most of the other relevant studies and challenged their findings [29]. Second, we adopted a validated instrument to assess EBP skills. This instrument was developed based on adult learning models, and covered the four key components of EBP. This tool was more comprehensive and practical than other scales which mainly focused on assessing the effects of curriculum on knowledge and skills [30], including subjective questionnaires and objective tools, such as the Fresno test [31], the Objective Structure Clinical Examination station [32], Berlin questionnaire [33], and the assessing competency of EBM tool [34]. Third, the study was sufficiently powered by including adequate numbers of participants according to the sample size estimation.

Some limitations of our study should also be noted. First, this study is not a RCT which may lead to selection bias and we only included two centers in this study for convenience sampling. This may limit the generalizability of our results to other settings in China [35]. Second, the EBM educational intervention applied was based on the curricular design by our university and teaching hospital. The curriculum tends to be more theoretical than practical. Further studies should pay more attention to more practical components, such as how to use evidence in practice and how to re-evaluate the evidence-based practice. The contents in future training should also be tailored to the audience according to their background and knowledge when the training program is designed and implemented. Third, long-term outcomes should have been assessed to investigate the duration of the interventions’ effects on clinical practice. Finally, the training duration, strategies for interactive response and feedback, as well as other details should be optimized in future implementation of face-to-face EBM training.

## Conclusion

In conclusion, a face-to-face EBM training course may improve EBP knowledge, attitudes, personal application and anticipated future use in primary healthcare professionals. Further studies will be required to confirm our results and to optimize the implementation of the face-to-face EBM training.

## Additional files

Additional file 1: Consent to participate “Evidence-Based Medicine Training” Project. (DOC 26 kb)

Additional file 2: Questionnaires of EBM skills. (DOC 28 kb)

## Abbreviations

EBM: Evidence-based Medicine
EBP: Evidenced-based practice
EBP-A: EBP-attitude
EBP-F: EBP-future anticipated use
EBP-K: EBP-knowledge
EBP-P: EBP-personal application
GPs: General Practitioners

## References

[1] Sackett DL, Rosenberg WMC, Gray JAM, Haynes RB, Richardson WS (1996). Evidence based medicine: what it is and what it isn't. BMJ.

[2] Dawes M, Summerskill W, Glasziou P, Cartabellotta A, Martin J, Hopayian K (2005). Sicily statement on evidence-based practice. BMC Med Educ.

[3] Kulier R, Gulmezoglu AM, Zamora J, Plana MN, Carroli G, Cecatti JG (2012). Effectiveness of a clinically integrated e-learning course in evidence-based medicine for reproductive health training: a randomized trial. JAMA.

[4] Prasad K (2012). Teaching evidence-based medicine in resource-limited countries. JAMA.

[5] Kortekaas MF, Bartelink ML, Boelman L, Hoes AW, de Wit NJ (2015). General practice trainees' information searching strategies for clinical queries encountered in daily practice. Fam Pract.

[6] Young T, Rohwer A, Volmink J, Clarke M (2014). What are the effects of teaching evidence-based health care (EBHC)? Overview of systematic reviews. PLoS One.

[7] Zwolsman S, te Pas E, Hooft L, Wieringa-de Waard M, van Dijk N (2012). Barriers to GPs' use of evidence-based medicine: a systematic review. Br J Gen Pract.

[8] Flores-Mateo G, Argimon JM (2007). Evidence based practice in postgraduate healthcare education: a systematic review. BMC Health Serv Res.

[9] Maloney S, Nicklen P, Rivers G, Foo J, Ooi YY, Reeves S (2015). A cost-effectiveness analysis of blended versus face-to-face delivery of evidence-based medicine to medical students. J Med Internet Res.

[10] Khan KS, Coomarasamy A (2006). A hierarchy of effective teaching and learning to acquire competence in evidenced-based medicine. BMC Med Educ..

[11] Shaneyfelt T, Baum KD, Bell D, Feldstein D, Houston TK, Kaatz S (2006). Instruments for evaluating education in evidence-based practice: a systematic review. JAMA.

[12] Aronoff SC, Evans B, Fleece D, Lyons P, Kaplan L, Rojas R (2010). Integrating evidence based medicine into undergraduate medical education: combining online instruction with clinical clerkships. Teach Learn Med.

[13] Argimon-Pallas JM, Flores-Mateo G, Jimenez-Villa J, Pujol-Ribera E (2011). Effectiveness of a short-course in improving knowledge and skills on evidence-based practice. BMC Fam Pract.

[14] Shuval K, Shachak A, Linn S, Brezis M, Feder-Bubis P, Reis S (2007). The impact of an evidence-based medicine educational intervention on primary care physicians: a qualitative study. J Gen Intern Med.

[15] Gruppen LD, Rana GK, Arndt TS (2005). A controlled comparison study of the efficacy of training medical students in evidence-based medicine literature searching skills. Acad Med.

[16] Ilic D, Nordin RB, Glasziou P, Tilson JK, Villanueva E (2015). A randomised controlled trial of a blended learning education intervention for teaching evidence-based medicine. BMC Med Educ..

[17] Mahmic-Kaknjo M, Kadic D, Hodzic H, Spahic-Sarajlic S, Hadzic E, Ademovic E (2015). Awareness, knowledge, use, and attitudes toward evidence based medicine in a developing country: survey of physicians in a canton in Bosnia and Herzegovina. Croat Med J.

[18] te Pas E, Wieringa-de Waard M, de Ruijter W, van Dijk N (2015). Learning results of GP trainers in a blended learning course on EBM: a cohort study. BMC Med Educ..

[19] Zwickey H, Schiffke H, Fleishman S, Haas M, Cruser d A, LeFebvre R (2014). Teaching evidence-based medicine at complementary and alternative medicine institutions: strategies, competencies, and evaluation. J Altern Complement Med.

[20] Barghouti F, Halaseh L, Said T, Mousa AH, Dabdoub A (2009). Evidence-based medicine among Jordanian family physicians: awareness, attitude, and knowledge. Can Fam Physician.

[21] Gorgon EJ, Basco MD, Manuel AT (2013). Teaching evidence based practice in physical therapy in a developing country: a national survey of Philippine schools. BMC Med Educ..

[22] Alahdab F, Firwana B, Hasan R, Sonbol MB, Fares M, Alnahhas I (2012). Undergraduate medical students’ perceptions, attitudes, and competencies in evidence-based medicine (EBM), and their understanding of EBM reality in Syria. Bmc Res Notes.

[23] Ma X, Xu B, Liu Q, Zhang Y, Xiong H, Li Y (2014). Effectiveness of evidence-based medicine training for undergraduate students at a Chinese military Medical University: a self-controlled trial. BMC Med Educ..

[24] Johnston JM, Leung GM, Fielding R, Tin KY, Ho LM (2003). The development and validation of a knowledge, attitude and behaviour questionnaire to assess undergraduate evidence-based practice teaching and learning. Med Educ.

[25] Rubenstein LV, Mittman BS, Yano EM, Mulrow CD (2000). From un- derstanding health care provider behavior to improving health care: the QUERI framework for quality improvement. Quality Enhancement Research Initiative. Med Care.

[26] Kok R, Hoving JL, Smits PBA, Ketelaar SM, van Dijk FJH (2013). A clinically integrated post-graduate training Programme in evidence-based medicine versus ‘no intervention’ for improving disability evaluations: a cluster randomised clinical trial. PLoS One.

[27] Shaneyfelt T, Baum KD, Bell D, Feldstein D, Houston TK, Kaatz S, Whelan C, Green M (2006). Instruments for evaluating education in evidence-based practice: a systematic review. JAMA.

[28] Roger E, Kreptul D (2015). Systematic review of evidence-based medicine tests for family physician residents[J]. Fam Med.

[29] Hatala R, Guyatt G (2002). Evaluating the teaching of evidence-based medicine. JAMA.

[30] Thomas RE, Kreptul D (2015). Systematic review of evidence-based medicine tests for family physician residents. Fam Med.

[31] Ramos KD, Schafer S, Tracz SM (2003). Validation of the Fresno test of competence in evidence based medicine. BMJ.

[32] Dong T, Swygert KA, Durning SJ, Saguil A, Gilliland WR, Cruess D (2014). Validity evidence for medical school OSCEs: associations with USMLE(R) step assessments. Teach Learn Med.

[33] Fritsche L, Greenhalgh T, Falck-Ytter Y, Neumayer HH, Kunz R (2002). Do short courses in evidence based medicine improve knowledge and skills? Validation of Berlin questionnaire and before and after study of courses in evidence based medicine. Bmj Br Med J.

[34] Ilic D, Nordin RB, Glasziou P, Tilson JK, Villanueva E (2014). Development and validation of the ACE tool: assessing medical trainees’ competency in evidence based medicine. BMC Med Educ..

[35] Cheng HM, Guo FR, Hsu TF, Chuang SY, Yen HT, Lee FY (2012). Two strategies to intensify evidence-based medicine education of undergraduate students: a randomised controlled trial. Ann Acad Med Singap.

