# Supplementary material for: Efficacy of evidence-based medicine training for primary healthcare professionals: a non-randomized controlled trial
Source: BMC Med Educ. 2018 Dec 7;18:299. doi: 10.1186/s12909-018-1404-y (PMC6286505; doi:10.1186/s12909-018-1404-y)
Supplement: Supplementary file 1 — Consent to participate “Evidence-Based Medicine Training” Project. (DOC 26 kb) [file 12909_2018_1404_MOESM1_ESM.doc]

**Additional file 1. Consent to participate “Evidence-Based Medicine Training” Project**

**Introduction:**

EBM empowers health care practitioners to recognize valid and clinically important evidence relevant to their practice. Also, EBM training should be evaluated and guided by evidence of its own effectiveness. Despite the increasing number of medical schools and postgraduate programs that have introduced EBM in their curricula, no data is available about the adoption of EBM by primary health care professionals in China. In fact, most of them haven’t yet accepted formal EBM education. Current evidence can only conclude that teaching EBM increases undergraduate medical students’ EBM knowledge, attitudes, personal application, and anticipated future use.

Thus, we need your participation. As a participant in my project, you will:

1) Group A will receive a 16-hour EBM course, including 2 lectures, 3 conferences and 3 small group discussions, and Group B will participate to the online self-learning model, take the same EBM course curriculum uploaded by a study assistant in advance in every week through the center’ s network.

2) Complete questionnaires consisted of 26 questions modified from a reliable assessment tool using a 6-point Likert scale before and after the intervention.

3)Any question on EBM learning can be offered to the teachers during the period.

Questionnaire results will be reported a group summary and not individually tracked. Participant responses will be anonymous. There are no physical or mental risks to your participation in the project.

Thank you for helping us in this special way,

Jiaojiao Fei, Yanhua Li

**Individual Consent Statement**

I agree to participate in the " Evidence-Based Medicine Training " project. I understand that any information I provide will be anonymous and not individually tracked. A summary of the project is available to me upon request.

Signature:

Date:
