# Supplementary material for: Efficacy of evidence-based medicine training for primary healthcare professionals: a non-randomized controlled trial
Source: BMC Med Educ. 2018 Dec 7;18:299. doi: 10.1186/s12909-018-1404-y (PMC6286505; doi:10.1186/s12909-018-1404-y)
Supplement: Supplementary file 2 — Questionnaires of EBM skills. (DOC 28 kb) [file 12909_2018_1404_MOESM2_ESM.doc]

**Additional files 2. Questionnaires of EBM skills**

*Evidence-based practice – knowledge*

Content: asking clinical questions, acquiring evidence, appraising evidence, applying evidence to a clinical situation, and assessing treatment effectiveness in terms of patient outcomes Question format: statements rated on a Likert scale (1 strongly disagree, 6 strongly agree)

1. Evidence-based medicine requires the use of critical appraisal skills to ensure the quality of all the research papers retrieved

2. Effective searching skills ⁄easy access to bibliographic databases and evidence sources are essential to practicing evidence-based medicine

3. Critically appraised evidence should be appropriately applied to the patient using clinical judgment and experience

4. The evidence-based medicine process requires the appropriate identification and formulation of clinical questions

5. Practicing evidence-based medicine increases the certainty that the proposed treatment is effective

*Attitudes toward evidence-based practice*

Content: perceived need for information, willingness to practice EBP, perceived role of EBP in clinical practice, attitude about EBP’s threat to clinical practice Question format: statements rated on a Likert scale (1 strongly disagree, 6 strongly agree)

1. If evidence-based medicine is valid, then anyone can see patients and do what doctors do

2. There is no reason for me personally to adopt evidence- based medicine because it is just a fad (or fashion ) that will pass with time

3. Evidence-based medicine is cook-book medicine that disregards clinical experience

4. Doctors, in general, should not practice evidence-based medicine because medicine is about people and patients, not statistics

5. Evidence-based medicine ignores the art of medicine

6. Previous work experience is more important than research findings in choosing the best treatment available for a patient

*Personal application and use of evidence-based practice*

Content: access and acquisition of evidence, application to patient care, barriers to adopting EBP, contribution of EBP to clinical reasoning and learning, current proportion of clinical activity based on EBP principles, frequency of actual use of EBP, perceived need for EBP each day or week and for each patient encounter, overall use of EBP in the past year (1 never, 5 every day; 1 not at all, 6 completely)

1. How frequently do you access medical evidence from a textbook?

2. How frequently to you access medical evidence in general?

3. How frequently do you access medical evidence on the Internet (excluding Medline and Cochrane Reviews)?

4. How frequently do you access medical evidence from original research papers?

5. How frequently do you access medical evidence from the Cochrane database?

6. How frequently do you access medical evidence from secondary sources such as the ACP Journal Club, the Journal of Evidence-Based Medicine, POEMs (Patient Oriented Evidence that Matters) or CATs (Critically Appraised Topics)?

*Future use of evidence-based practice*

Content: perceived future importance of EBP to medical practice, willingness to practice EBP in the future, usefulness of EBP in the future, potential barriers to the adoption of EBP currently and in the future. Question format: statements rated on a Likert scale (1 very unwilling, 6 very willing; 1 completely useless, 6 very useful; 1 not at all, 6 completely)

1. Compared to 1 year ago, how useful do you believe evidence-based medicine will be in your future practice as a doctor?

2. Compared to 1 year ago, how willing are you to practice evidence-based medicine as a doctor in the future?

3. You personally appreciate the advantages of practicing evidence-based medicine

4. Evidence-based medicine should be an integral part of the undergraduate medical curriculum

5. Compared to 1 year ago, how much do you support lifelong learning using evidence-based medicine techniques?

Annals Academy of Medicine

6. Compared to 1 year ago, how much do you support the principles of evidence-based medicine?

7. How much do you consider the practice of evidence- based medicine a routine part of your learning?

8. How much has the practice of evidence-based medicine changed the way you learn?

9. How easy or difficult has it been for you to practice evidence-based medicine as a medical student in the last month?
